# Natural Autoantibodies Negatively Correlate with Hepatocellular Carcinoma Incidence in Cirrhosis

**DOI:** 10.1158/2767-9764.CRC-26-0007

**Published:** 2026-05-15

**Authors:** Manaf Alsudaney, Ashley Kim, Rami Hemadeh, Naomy Kim, Daniel Legaspi, Osama Khattab, Jessica Liu, Ekaterina K. Koltsova, Hasmik Adetyan, Tamar Yalda, Hirsh Trivedi, Walid S. Ayoub, Ju Dong Yang

**Affiliations:** 1 Karsh Division of Gastroenterology and Hepatology, Department of Medicine, Cedars-Sinai Health Sciences University, Los Angeles, California.; 2 Comprehensive Transplant Center, Cedars-Sinai Health Sciences University, Los Angeles, California.; 3 Chicago Medical School, Rosalind Franklin University of Medicine and Science, North Chicago, Illinois.; 4 Department of Medicine, Cedars-Sinai Cancer, Cedars-Sinai Health Sciences University, Los Angeles, California.; 5 Department of Cardiology, Cedars-Sinai Cancer, Cedars-Sinai Health Sciences University, Los Angeles, California.; 6 Department of Biomedical Sciences, Cedars-Sinai Cancer, Cedars-Sinai Health Sciences University, Los Angeles, California.; 7 Division of Gastroenterology and Hepatology, Dell Medical School, University of Texas at Austin, Austin, Texas.; 8 Samuel Oschin Comprehensive Cancer Institute, Cedars-Sinai Health Sciences University, Los Angeles, California.

## Abstract

Hepatocellular carcinoma (HCC) remains a leading cause of cancer death, and existing HCC risk stratification models in cirrhosis poorly identify patients at the highest risk. We evaluated whether baseline antinuclear antibody (ANA) status is independently associated with incident HCC and whether adding ANA improves risk prediction. We conducted a single-center, retrospective cohort of adults (age ≥18 years) with established cirrhosis and clinically obtained ANA testing within 12 months of cirrhosis diagnosis. Follow-up began at the cirrhosis index date; the primary outcome was incident HCC. Associations were assessed using cause-specific Cox models with prespecified clinical covariates. We compared discrimination for a clinical model with and without ANA and performed inverse-probability–weighted and immunologic (IgG/neutrophil-to-lymphocyte ratio) sensitivity analyses in complete cases. Among 1,023 participants [mean age 53.7 years (SD 10.9); 57% male], 102 developed incident HCC over a median 4.7 years of follow-up. HCC incidence was lower in ANA-positive versus ANA-negative patients (1.10 vs. 3.73 per 100 person-years; log-rank *P* < 0.001). ANA positivity was associated with lower HCC risk in multivariable models (HR, 0.32; 95% CI, 0.20–0.52). Adding ANA improved discrimination (C-index 0.727–0.750) and model fit (likelihood-ratio *P* < 0.001). Findings were consistent in weighted and laboratory-complete sensitivity analyses. In cirrhosis, baseline ANA positivity is independently associated with lower incident HCC risk and provides incremental prognostic information beyond routine clinical factors. Prospective validation and mechanistic studies are warranted.

**Significance:**

Natural autoantibodies (ANA) are routinely measured, inexpensive, and widely accessible, yet their prognostic value in cirrhosis is not used for HCC risk stratification. In this retrospective cohort, baseline ANA positivity was independently associated with lower incident HCC risk and improved model discrimination beyond clinical factors. These findings support repurposing a common clinical test to refine individualized surveillance intensity and motivate prospective validation and mechanistic studies.

## Introduction

Hepatocellular carcinoma (HCC) occurs in the setting of chronic liver injury and inflammation and is now among the most rapidly rising causes of cancer mortality (1). Despite guidelines advocating for HCC surveillance in cirrhosis, HCC detection at an early and curable stage remains suboptimal. Current clinical risk models have modest discrimination, especially in the growing population with metabolic dysfunction–associated steatotic liver disease (MASLD; ref. 2). MASLD has become a leading driver of cirrhosis and HCC, and HCC risk in patients with cirrhosis with metabolic dysfunction is highly heterogeneous, contributing to the limited performance of one-size-fits-all surveillance strategies (3). There is an urgent need for scalable markers that identify patients who remains at the highest risk and who would benefit from intensified surveillance or preventive strategies.

Autoantibodies are typically framed as epiphenomena (diagnostic markers of autoimmune conditions or collateral participants in chronic inflammation; refs. 4–6). They occur when a loss of immune tolerance leads to antibody generation against self-antigens (autoimmunity; refs. 7, 8). Whereas this breakdown can drive autoimmune disease and has been linked to a broad spectrum of conditions, including cancers, autoantibodies are also detectable in a substantial proportion of healthy individuals (9). Yet a parallel immunology literature suggests that “natural” or inducible autoantibodies may participate in immunosurveillance by recognizing altered self on dysplastic or immune-evasive cells that escape classic T cell–mediated detection because of self-tolerance (10–12).

In the liver, in which chronic injury alters antigen presentation and remodels the tumor microenvironment, such humoral signals could possibly arise before clinically detectable HCC (13, 14), and function as an early warning system rather than just byproducts of autoimmunity.

Our team’s prior work links chronic inflammation, immune surveillance, and iatrogenic immunosuppression with elevated HCC risk (15), including observations around prolonged prednisone exposure and hepatocarcinogenesis in susceptible hosts (16). Building on this foundation, supported by prior animal-based works (17, 18), we hypothesized that baseline autoantibody positivity, principally antinuclear antibodies (ANA) would be associated with lower HCC incidence by marking an active antitumor humoral response. Alternatively, if autoantibodies were simply a surrogate for injurious inflammation, we would expect higher HCC risk. Investigating these opposing predictions may clarify how autoimmunity and inflammation contribute to hepatocarcinogenesis.

We assembled a large single-center retrospective cohort of adults with cirrhosis, abstracting autoantibody results near the time of cirrhosis diagnosis and tracking incident HCC. We prespecified time-to-event analyses adjusting for major confounders, with sensitivity analyses excluding early events to mitigate reverse causality. To move beyond association, we examined whether incorporating autoantibody data into standard clinical models improves discrimination, thereby assessing potential clinical utility.

We therefore aimed to determine whether baseline autoantibody positivity (primarily ANA) is independently associated with incident HCC in cirrhosis and to quantify the incremental predictive value of ANA information beyond standard clinical factors.

## Materials and Methods

### Study design

We conducted a single-center, retrospective cohort study at Cedars-Sinai Medical Center. Adults with cirrhosis evaluated between January 2005 and December 2024 were followed longitudinally. The study was Institutional Review Board (IRB)-approved (STUDY00004026) with a waiver of consent. Reporting follows Strengthening the Reporting of Observational Studies in Epidemiology (STROBE) guidelines.

### Cohort identification

We identified potentially eligible adults via diagnosis, procedure, and pathology codes and problem list terms indicative of cirrhosis, using DEEP 6 cohort builder, followed by chart review confirmation. DEEP 6 was used as a broad, high-sensitivity case-finding tool to avoid missing potentially eligible patients with cirrhosis. We then performed structured chart review to confirm cirrhosis and apply study eligibility criteria. Among 1,829 patients with confirmed cirrhosis after chart review, 1,023 had clinically obtained ANA testing within the prespecified exposure window and met follow-up and outcome criteria and were included in the analytic cohort; 806 were excluded from the primary analysis because ANA testing was not available.

Inclusion criteria were age ≥18 years, clinically or histologically confirmed cirrhosis, and availability of a baseline ANA test within a prespecified exposure window relative to cirrhosis diagnosis. The index date was defined as the date cirrhosis was first established in the Cedars-Sinai record or, for patients with a prior outside diagnosis, the earliest date on which that diagnosis was explicitly confirmed in Cedars-Sinai documentation. Historic diagnosis dates were accepted only if not earlier than January 1, 2005. Inclusion criteria additionally required at least one confirmatory imaging or histology assessment of HCC status occurring ≥6 months after index; records lacking such follow-up were excluded. ANA measurements obtained within 12 months before the index date (or, if available, between the index date and the outcome event) were eligible; when multiple results fell within this window, we used the value closest to the index date. ANA exposure was analyzed as status [positive vs. negative, with positivity defined on indirect immunofluorescence (IFA) at ≥1:40 in adults] and as ordered titer categories negative (<1:40), low-positive (1:40–1:80), and high-positive (≥1:160), consistent with hepatology guideline recommendations that positive sera be titrated and that ≥1:40 represents a significant adult titer (19).

The ANA exposure window was selected because ANA status is relatively stable over time, most repeat ANA tests separated by ≥1 year are unchanged, so baseline misclassification from using the closest in window result is unlikely (20). We selected a 12-month window *a priori* to balance temporal proximity to cohort entry with feasibility in real-world testing patterns. A shorter window would reduce sample size and precision, whereas a longer window could increase exposure misclassification due to changes in immune status over time.

For ANA-positive tests, we additionally abstracted the reported IFA staining pattern (e.g., speckled, homogeneous, nucleolar, centromere, and nuclear envelope/rim) when available in the laboratory report.

Our original plan for the autoantibody exposure was to evaluate a broad panel [ANA, smooth muscle antibody (SMA), antimitochondrial antibody, and anti-dsDNA]. In practice, ANA was present for most participants, whereas non-ANA markers were rarely and inconsistently obtained outside of specific indications, precluding powered analyses in the overall cohort. Because non–organ-specific autoantibodies, including ANA and SMA, are detected in a substantial subset of patients with MASLD, although their prognostic significance is unclear (21, 22), we additionally prespecified an exploratory evaluation of SMA restricted to the MASLD cirrhosis subset with available SMA results, because event counts were limited within etiologic subgroups, MASLD- and SMA-restricted models were prespecified as exploratory/hypothesis-generating.

SMA titers were reported as “<20,” “negative,” 1:20, 1:40, 1:80, 1:160, 1:320, or 1:640, with a reference range of <20. Accordingly, SMA positivity was defined as titers ≥1:20, whereas values <20 (including “negative”) were considered SMA-negative. Non-ANA markers such as SMA and antimitochondrial antibody were obtained selectively in clinical practice and were therefore evaluated only in an exploratory MASLD-restricted analysis.

In a prespecified exploratory analysis, we restricted the cohort to participants with MASLD cirrhosis and repeated the primary time-to-HCC analyses. ANA titer was categorized as negative (<1:40) versus positive (≥1:40), as in the main analysis. Within the MASLD subset, we estimated HCC incidence rates per 100 person-years by ANA status with exact Poisson 95% confidence intervals (CI) and fit Cox proportional hazards models with time zero defined as the index date of cirrhosis diagnosis and censoring at liver transplantation, death, loss to follow-up, or last clinical encounter. Multivariable models adjusted for age (per 10 years), sex, model for end-stage liver disease (MELD) ≥15, Child–Turcotte–Pugh (CTP) class B/C, AFP ≥20 ng/mL, chronic prednisone exposure, and race/ethnicity (Hispanic White, non-Hispanic Asian, non-Hispanic Black, non-Hispanic other vs. non-Hispanic White).

Because SMA testing was performed predominantly in MASLD, we further restricted to patients with MASLD with an SMA result to explore whether SMA positivity (titer ≥1:20, per laboratory reference) predicted HCC. We estimated HCC incidence rates and Cox models comparing SMA-positive versus SMA-negative patients, with and without adjustment for ANA status and the same clinical covariates. Given the limited number of events, these SMA analyses were considered hypothesis-generating.

We excluded ANA results first appearing only after an HCC diagnosis to align with the chronologic ordering hypothesis and minimize reverse causation (protopathic bias; ref. 23). We also excluded individuals with known HCC before or at cohort entry, prior liver transplantation before index, insufficient documentation to establish cirrhosis or outcomes, or fewer than 6 months of at-risk time without subsequent outcome ascertainment. Of 2,832 DEEP 6 cohort builder identified adults, 1,023 contributed to the primary time-to-event analysis.

### Outcome design

The primary outcome was incident HCC after cohort entry. To minimize misclassification of nonevents, we classified participants as HCC-free only if they had at least one clinical or imaging assessment ≥6 months after the index date documenting no evidence of HCC. All HCC diagnoses were adjudicated by a multidisciplinary tumor board based on histopathology and/or imaging consistent with contemporary AASLD criteria (24). Follow-up accrued from index until the earliest of HCC diagnosis or a final clinical/imaging assessment confirming no HCC. Patients who underwent liver transplantation or died without HCC were censored at the date of the last assessment confirming no HCC prior to transplant or death. Secondary outcomes were prespecified to align with clinical interpretation and the data structure: HCC incidence rates per 100 person-years overall and stratified by ANA status and by ANA titer, with exact Poisson 95% CIs calculated using the classic Garwood approach (25).

### Statistical analysis

We analyzed the time to incident HCC from the date of cirrhosis diagnosis (index). Autoantibody exposure was operationalized as ANA-positive (titer ≥1:40) versus -negative (<1:40). In secondary analyses, ANA titer was modeled as an ordered categorical variable negative (<1:40), low-positive (1:40–1:80), and high-positive (≥1:160). Baseline characteristics by ANA were summarized using means with standard deviations (SD) or proportions, and we reported standardized mean differences to convey the magnitude of any between-group imbalance rather than relying on null-hypothesis testing.

We estimated unadjusted HCC incidence rates per 100 person-years with exact (Garwood) 95% CIs and compared cumulative incidence using Kaplan–Meier (KM) curves with a log-rank test. For multivariable inference, we fit cause-specific Cox proportional hazards models to estimate hazard ratios (HR) and 95% CIs for the association of ANA with incident HCC, treating liver transplantation and non-HCC death as censoring events because our primary objective was to evaluate etiologic associations with incident HCC rather than cumulative incidence in the presence of competing risks. Prespecified covariates included age (per 10 years; ref. 26), sex, MELD, Child–Pugh class, α-fetoprotein [AFP; dichotomized at 20 ng/mL for modeling; summarized as median (IQR)], chronic prednisone use, race/ethnicity (Hispanic White, non-Hispanic Asian, non-Hispanic Black, non-Hispanic other vs. non-Hispanic White), and cirrhosis etiology [hepatitis B virus (HBV), hepatitis C virus (HCV), MASLD, alcohol-associated liver disease (ALD), autoimmune (autoimmune hepatitis/primary biliary cholangitis/primary sclerosing cholangitis), and multifactorial/other; reference MASLD]. Multifactorial etiology denotes >1 documented contributing etiology (e.g., MASLD plus ALD and/or viral hepatitis). Given known higher HCC risk with viral hepatitis and multifactorial causes relative to autoimmune etiologies, etiology adjustment was prespecified. No formal *a priori* power calculation was performed because cohort size was determined by available records and eligibility criteria; to reduce overfitting given 102 HCC events, we prespecified a parsimonious covariate set and limited degrees of freedom consistent with conventional events-per-parameter guidance; MASLD etiology was based on clinician documentation in the electronic health record. MELD and CTP index liver dysfunction severity that can influence both hepatocarcinogenesis and surveillance patterns, so we adjusted for them to mitigate confounding by disease stage and care intensity (16). AFP ≥20 ng/mL was included as a tumor-biology proxy and because a 20 ng/mL threshold is widely used in guideline-based surveillance/recall algorithms (17). Chronic prednisone indicates systemic immunosuppression and was included *a priori* given our prior findings relating long-term corticosteroid exposure to HCC risk (8). Finally, etiology was grouped because HCC risk varies meaningfully across viral, metabolic, alcohol-related, and immune-mediated liver disease (15). Confounding by indication and disease severity was handled via prespecified covariate adjustment and subgroup/interaction checks, prespecified subgroup analyses evaluated whether the ANA–HCC association varied by sex, ANA with prolonged prednisone use (27), and etiology group [autoimmune vs. nonautoimmune (HBV/HCV/MASLD/ALD/other)]. We fit stratified Cox models and tested multiplicative interaction terms (ANA × etiology group; ANA × sex; ref. 27). Potential surveillance bias was explored by describing imaging frequency patterns and, in a sensitivity analysis, restricting to participants with regular surveillance intervals (28). Misclassification was reduced through multidisciplinary outcome adjudication and by excluding ANA results obtained after, or within, the peridiagnostic window of HCC. We assessed proportional hazards with Schoenfeld-type diagnostics and visual inspection of log-minus-log plots; no material violations were detected.

As a sensitivity analysis to address selection related to clinically obtained ANA testing, we modeled the probability of ANA testing using demographics available in the full confirmed-cirrhosis cohort and applied inverse-probability weights in the primary Cox model.

To further reflect host immune status, we conducted prespecified sensitivity models adding IgG (per SD increase) and an inflammatory index [natural-log of the neutrophil-to-lymphocyte ratio (NLR), per SD; ref. 18], sensitivity analyses addressed immunologic covariates. Because IgG and the NLR were intermittently missing, we conducted complete-case models that added standardized IgG (*z*-score) and log-transformed NLR (also *z*-scored). To retain stability with variable missingness, we used an adaptive approach that prioritized inclusion of both markers when possible and defaulted to age + IgG when log (NLR) would have excessively restricted the sample; final model composition and complete-case counts are reported alongside results.

To quantify prognostic utility, we summarized discrimination with Harrell’s C-index for a base clinical model (age, sex, MELD, CTP, AFP, prednisone, race/ethnicity, and etiology) and for the same model augmented with ANA. Nested models (±ANA) were compared with the likelihood-ratio test; Akaike information criterion (AIC) and Bayesian information criterion (BIC) were also reported. Prespecified subgroups (autoimmune vs. nonautoimmune etiologies; women vs. men) were evaluated with stratified Cox models and multiplicative interaction terms. All tests were two-sided with α = 0.05. Analyses were performed in Python; figures/tables were generated directly from the analysis outputs.

### Ethics approval and consent to participate

This retrospective cohort study was approved by the Cedars-Sinai Medical Center IRB (STUDY00004026), which granted a waiver of informed consent because of the minimal-risk design and use of deidentified data.

### Reporting guideline

This study adhered to the STROBE guidelines for cohort studies.

## Results

We identified 1,829 patients with confirmed cirrhosis after chart review; 1,023 had clinically obtained ANA testing within the prespecified exposure window and comprised the analytic cohort.

Across the 1,023 adults, there were 102 incident HCC events during a median follow-up of 4.7 years. HCC incidence was 1.10 versus 3.73 per 100 person-years for ANA+ versus ANA−, respectively. Cumulative incidence (1 − KM) curves separated early and remained distinct (log-rank *Z* = −5.42, *P* < 0.001). Baseline features by ANA status are summarized in Table 1. Detailed unadjusted and adjusted cause-specific Cox model estimates are shown in Table 2.

**Table 1.** Baseline characteristics by ANA.

Variable	ANA− (*n* = 506)	ANA+ (*n* = 517)	SMD
Age, years	53.99 ± 11.07	53.46 ± 10.74	0.049
Male, *n* (%)	295 (58.3%)	292 (56.5%)	0.037
MELD	18.21 ± 9.26	17.76 ± 8.47	0.050
MELD ≥15, *n* (%)	287 (56.7%)	307 (59.4%)	0.054
CTP score	8.19 ± 2.44	8.29 ± 2.43	0.042
CTP B/C, *n* (%)	349 (69%)	359 (69.4%)	0.010
AFP, ng/mL [median (IQR)]	4 (2–6)	3 (2–5)	—
AFP ≥20 ng/mL, *n* (%)	23 (4.5%)	11 (2.1%)	0.135
Chronic prednisone, *n* (%)	32 (6.3%)	52 (10.1%)	0.136
Race/ethnicity	​	​	​
Hispanic White, *n* (%)	218 (43.1%)	229 (44.3%)	0.024
Non-Hispanic White, *n* (%)	185 (36.6%)	193 (37.3%)	0.016
Non-Hispanic Black, *n* (%)	26 (5.1%)	26 (5%)	0.005
Non-Hispanic Asian, *n* (%)	34 (6.7%)	31 (6%)	0.030
Non-Hispanic other, *n* (%)	43 (8.5%)	38 (7.4%)	0.043
Etiology	​	​	​
HBV, *n* (%)	15 (3%)	7 (1.4%)	0.111
HCV, *n* (%)	45 (8.9%)	41 (7.9%)	0.035
MASLD, *n* (%)	128 (25.3%)	116 (22.4%)	0.067
ALD, *n* (%)	206 (40.7%)	200 (38.7%)	0.041
Autoimmune, *n* (%)	28 (5.5%)	45 (8.7%)	0.124
Multifactorial, *n* (%)	40 (7.9%)	58 (11.2%)	0.113
Other, *n* (%)	44 (8.7%)	50 (9.7%)	0.034

Data are presented as the mean ± SD, median (IQR), or *n* (%). Percentages use the total number in each ANA group as the denominator. Missing data for baseline covariates were low: MELD, 7/1,023 (0.7%); CTP, 4/1,023 (0.4%); AFP, 45/1,023 (4.4%); age, sex, race/ethnicity, etiology, and chronic prednisone use had no missing values. Multifactorial etiology denotes >1 documented contributing etiology (e.g., MASLD plus ALD and/or viral hepatitis).

Abbreviation: SMD, Standardized mean difference.

**Table 2.** Association of baseline variables with incident HCC in cause-specific Cox models.

Variable	Unadjusted HR (95% CI)	*P* value	Adjusted HR^a^ (95% CI)	*P* value
ANA-positive (≥1:40)	0.30 (0.19–0.48)	<0.001	0.32 (0.20–0.52)	<0.001
Age (per 10 years)	2.11 (1.63–2.73)	<0.001	2.36 (1.77–3.16)	<0.001
Male sex	1.34 (0.89–2.01)	0.157	1.35 (0.86–2.12)	0.188
MELD ≥15 (vs. <15)	1.29 (0.87–1.91)	0.200	1.67 (1.01–2.77)	0.045
CTP B/C (vs. A)	0.95 (0.64–1.42)	0.806	1.09 (0.66–1.82)	0.728
AFP ≥20 ng/mL (vs. <20)	2.31 (1.28–4.17)	0.006	1.34 (0.69–2.63)	0.391
Chronic prednisone use	0.69 (0.32–1.49)	0.344	1.02 (0.44–2.34)	0.968
Race/ethnicity	​	​	​	​
Hispanic White vs. non-Hispanic White	1.03 (0.68–1.58)	0.879	1.12 (0.72–1.76)	0.606
Non-Hispanic Asian vs. non-Hispanic White	1.14 (0.57–2.29)	0.717	0.74 (0.34–1.65)	0.466
Non-Hispanic Black vs. non-Hispanic White	0.88 (0.31–2.47)	0.806	0.65 (0.22–1.96)	0.443
Non-Hispanic other vs. Non-Hispanic White	0.31 (0.08–1.28)	0.106	0.37 (0.09–1.55)	0.172
Etiology	​	​	​	​
ALD vs. MASLD	0.70 (0.41–1.19)	0.186	0.89 (0.49–1.61)	0.693
Autoimmune vs. MASLD	0.40 (0.15–1.04)	0.059	0.81 (0.29–2.28)	0.684
HBV vs. MASLD	1.49 (0.61–3.66)	0.381	1.76 (0.57–5.41)	0.327
HCV vs. MASLD	1.37 (0.78–2.42)	0.278	1.60 (0.85–2.98)	0.144
Multifactorial vs. MASLD	0.66 (0.30–1.46)	0.305	1 (0.43–2.31)	0.998
Other vs. MASLD	0.96 (0.42–2.22)	0.929	0.89 (0.39–2.07)	0.779

a Unadjusted HRs are from separate Cox models for each covariate. Adjusted HRs are from a single multivariable cause-specific Cox model including all listed covariates (*n* = 1,023; 102 HCC events). ANA-positive is defined as titer ≥1:40; age is modeled per 10-year increase; reference categories are MELD <15, CTP class A, AFP <20 ng/mL, no chronic prednisone use, non-Hispanic White race/ethnicity, and MASLD etiology. Multifactorial etiology denotes >1 documented contributing etiology (e.g., MASLD plus ALD and/or viral hepatitis).

Among ANA-positive patients, the most common IFA pattern was nuclear speckled (73%), followed by homogeneous (7%) and nucleolar (5%) patterns; nuclear envelope (membranous) and other patterns were rare.

**Figure 1.**
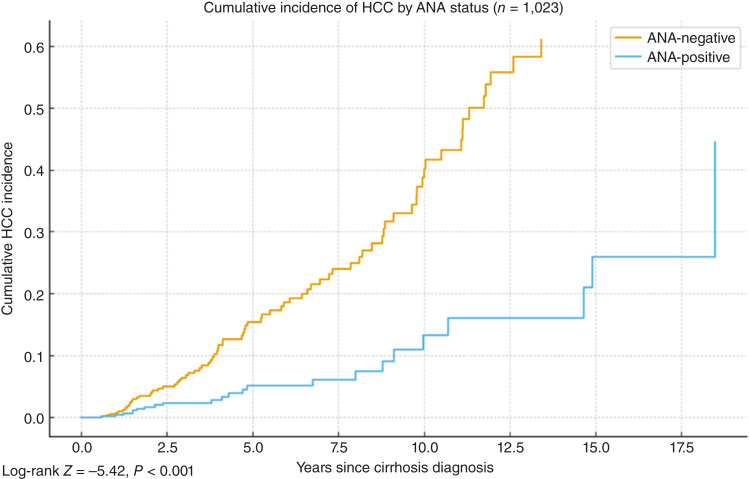
Cumulative incidence of HCC from cirrhosis diagnosis by ANA status.

**Figure 2.**
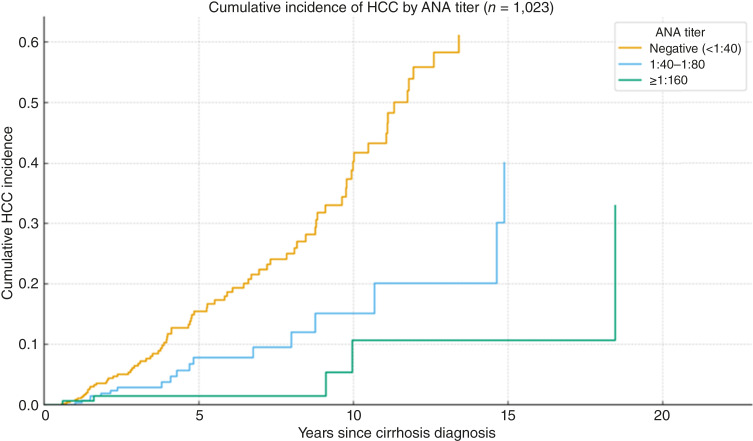
Cumulative incidence of HCC by baseline ANA titer. ANA titers were grouped as negative (<1:40), 1:40–1:80, and ≥1:160; higher titers show progressively lower HCC incidence. Log-rank test across titer groups: *χ*^2^ = 31.16 (df = 2), *P* < 0.001.

**Figure 3.**
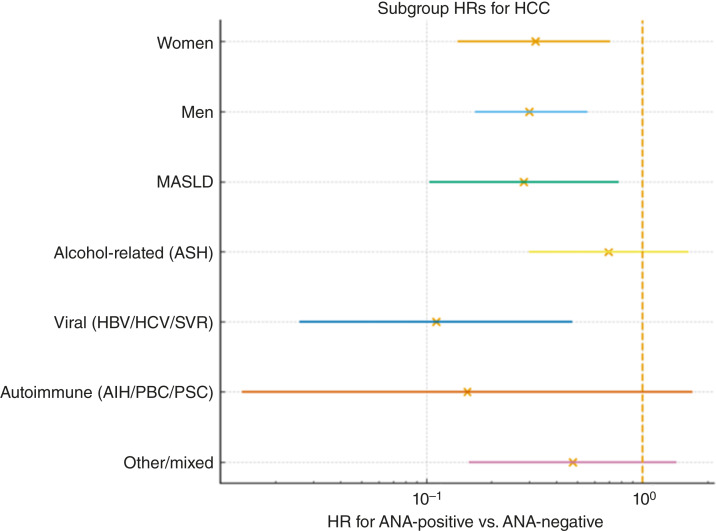
Adjusted HRs for HCC by prespecified subgroups. Adjusted HRs for HCC by prespecified subgroups (HRs with 95% CIs; vertical line at HR = 1). Subgroup models estimate the association of ANA positivity (titer ≥1:40) with HCC risk within sex and etiology strata [MASLD, alcohol-related (ASH), viral [HBV/HCV/sustained virologic response (SVR)], autoimmune (AIH/PBC/PSC), and other/mixed etiologies], adjusting for age, MELD, and Child–Pugh class within each stratum. AIH, autoimmune hepatitis; PBC, primary biliary cholangitis; PSC, primary sclerosing cholangitis.

HCC incidence was lower among participants who were ANA-positive at index than among those who were ANA-negative (1.10 vs. 3.73 per 100 person-years; Fig. 1). A visualization by ordered ANA titer is provided in Fig. 2, and subgroup estimates are shown in Fig. 3. When titers were grouped as negative (<1:40), 1:40 to 1:80, and ≥1:160, HCC incidence decreased stepwise across these categories, with the lowest rates observed in the ≥1:160 group (Table 3).

**Table 3.** Incidence rates by ANA and by titer.

ANA status	Events	Person-years	Rate per 100 person-years	CI (low)	CI (high)
Negative	79	2,120.167	3.726	2.95	4.64
Positive	23	2,085.250	1.103	0.70	1.66

ANA titer cat	Events	Person-years	Rate per 100 person-years	CI (low)	CI (high)
Negative (<1:40)	79	2,120.167	3.726	2.95	4.64
1:40–1:80	18	1,272.583	1.414	0.84	2.24
≥1:160	5	812.667	0.615	0.20	1.44

Person-years were calculated as follow-up months divided by 12. Rates and 95% CIs were calculated using exact Poisson (Garwood) methods and are expressed per 100 person-years. ANA titer categories were defined as negative (<1:40), low-positive (1:40–1:80), and high-positive (≥1:160).

In multivariable Cox models including age (per 10 years), sex, MELD ≥15, Child–Pugh class B/C, baseline AFP ≥20 ng/mL, chronic prednisone use, and race/ethnicity, ANA positivity was associated with lower HCC risk (HR, 0.32; 95% CI, 0.20–0.52). Age (per 10 years HR, 2.36; 95% CI, 1.77–3.16) and MELD ≥15 (HR, 1.67; 95% CI, 1.01–2.77) were associated with increased risk of HCC (Table 2).

To address selection related to clinically obtained ANA testing, baseline demographics of confirmed cirrhosis patients with versus without ANA testing are summarized in Supplementary Table S1. In an inverse-probability–weighted sensitivity analysis reweighting the analytic cohort to the full confirmed-cirrhosis cohort using available demographics, the association between ANA positivity and lower incident HCC risk was essentially unchanged (inverse-probability weighted HR, 0.32; 95% CI, 0.20–0.52; Supplementary Table S2).

To evaluate prognostic contribution of ANA, we first specified this clinical model as the baseline model and then compared its discrimination with the same model augmented with ANA. Adding ANA to a prespecified clinical model improved discrimination (C-index 0.727→0.750; ΔC = +0.024) and model fit (LR *χ*^2^ = 25.4, *P* = 4.65 × 10^−7^). AIC and BIC both decreased versus the clinical model. The inverse association of ANA positivity with HCC was present in nonautoimmune liver disease (HR, 0.32; 95% CI, 0.20–0.53). When we further stratified by specific etiologies [MASLD, alcohol-related cirrhosis, viral hepatitis [HBV/HCV/sustained virologic response (SVR)], autoimmune cholestatic/hepatitic disease, and other/mixed etiologies], ANA positivity was associated with lower HCC risk across all subgroups, with point estimates consistently below 1 (Fig. 3).

In prespecified exploratory analyses focused on MASLD cirrhosis and SMA, among 244 participants with MASLD cirrhosis (26 HCC events; 968.8 person-years of follow-up; median follow-up 2.8 years, IQR 1.8–5.2), ANA positivity remained associated with lower HCC risk. HCC incidence was 4.1 versus 1.1 per 100 person-years for ANA-negative and ANA-positive patients, respectively [incidence-rate ratio (IRR) 0.27; 95% CI, 0.10–0.72]. In MASLD-restricted Cox models, ANA positivity was associated with a lower hazard of HCC (unadjusted HR, 0.26; 95% CI, 0.10–0.69); this association persisted after adjustment for age, sex, MELD, CTP class, AFP, prednisone, and race/ethnicity (adjusted HR, 0.28; 95% CI, 0.10–0.77). The corresponding HCC-free survival curves for the MASLD subgroup are shown in Supplementary Fig. S1. In the MASLD subset with SMA testing (*n* = 206; 21 HCC events), HCC incidence was similar in SMA-negative and SMA-positive patients (2.46 vs. 2.87 per 100 person-years; IRR 1.17; 95% CI, 0.48–2.82). SMA positivity (defined as titer ≥1:20) was not clearly associated with HCC risk in unadjusted models (HR, 1.14; 95% CI, 0.47–2.76) or in multivariable models adjusting for ANA and clinical covariates (adjusted HR, 1.19; 95% CI, 0.41–3.46), although estimates were imprecise because of the small number of events (Supplementary Table S3).

Sensitivity analyses addressed immunologic covariates. Because NLR values were more frequently missing than differential counts needed to compute NLR, we used an adaptive complete-case approach. In the model adding age and standardized IgG (with log-transformed NLR included when available), the association of ANA with lower risk persisted; for the age + IgG complete-case subset (*n* = 807; 80 events), the HR for ANA positivity was 0.43 (95% CI, 0.25–0.73; *P* = 0.0018). In a sensitivity analysis subset with complete laboratory data including differential counts (*n* = 766; 75 events), further adjustment for log-transformed NLR log yielded an HR for ANA positivity of 0.45 (95% CI, 0.26–0.77), essentially unchanged from the main model, whereas log (NLR) itself was not independently associated with HCC (HR, 1.13; 95% CI, 0.87–1.46). Incidence summaries are provided in Table 3, and model coefficients are provided in Table 2 and Supplementary Tables S2–S4. IgG was missing in 21% of the analytic cohort (216/1,023), whereas differential counts to compute NLR were missing in 2% (20/1,023); therefore, complete-case models primarily reduced sample size due to IgG availability.

We performed bootstrap internal validation (1,000 resamples) of the base clinical Cox model and of the ANA-augmented model. Apparent C-indices were 0.727 for the base model and 0.750 for the model including ANA. After correction for optimism, C-indices were 0.71 to 0.72 (base) and 0.73 to 0.74 (base + ANA), respectively, yielding an optimism-corrected ΔC of approximately +0.015 to +0.025. Thus, bootstrap validation indicates minimal overfitting and preservation of the incremental discrimination gained by adding ANA, supporting the robustness of the primary findings.

## Discussion

In this retrospective cohort of adults with cirrhosis, baseline ANA positivity was associated with a lower subsequent hazard of incident HCC after adjustment for demographic factors, liver disease severity, AFP, etiology, and prednisone exposure. KM curves separated early and remained apart, consistent with durable differences in risk over follow-up. Adding ANA to a clinical model improved discrimination, supporting incremental prognostic information beyond standard risk factors. These findings support ANA as a candidate adjunctive risk marker and warrant external validation in cohorts with protocolized testing.

If baseline autoantibody status meaningfully stratifies HCC risk, the implications are practical: a widely available, low-cost ANA test could complement routine clinical factors to refine surveillance intensity. Conversely, if autoantibody positivity signals higher risk in other settings, this would support autoimmunity/chronic inflammation as carcinogenic drivers and motivate careful attention to modifiable inflammatory pathways and immunosuppression exposure.

Discrimination analyses showed that adding ANA to routine clinical factors improved C-index, supporting incremental clinical utility beyond association. Bootstrap internal validation showed minimal optimism and preservation of the incremental gain in C-index, supporting the robustness of the primary findings and its role as an adjunctive risk-stratification marker.

We prespecified ≥1:40 as the primary ANA threshold (adult laboratory positivity). When titers were grouped as negative (<1:40), 1:40 to 1:80, and ≥1:160, HCC incidence showed a graded decrease across these categories, with the lowest rates in the ≥1:160 group (Table 3), consistent with a dose–response pattern.

In the rapidly expanding MASLD population, risk-stratified HCC surveillance remains a major unmet need. In our MASLD-restricted analysis, ANA positivity remained independently associated with a substantially lower hazard of HCC, with effect estimates similar to those observed in the overall cirrhosis cohort. This suggests that ANA-linked antitumor humoral activity may be relevant even in metabolic cirrhosis, in which existing risk models have shown limited discrimination. By contrast, SMA, measured predominantly in patients with MASLD and defined as positive at titers ≥1:20 per our laboratory reference, was not clearly associated with HCC risk; HCC incidence rates were similar in SMA-negative and SMA-positive groups, and CIs were wide. These exploratory findings support ANA as the dominant autoantibody signal for HCC risk in this dataset and highlight the need for larger, prospective MASLD studies with systematic, protocolized autoantibody testing.

Not all immune-related markers aligned with ANA directionally, suggesting that ANA positivity should not be interpreted as a general proxy for systemic inflammatory activity. In this cohort, ANA positivity was associated with lower incident HCC risk, whereas total IgG (per SD) was associated with higher risk and NLR was not independently associated. Because these measures reflect different dimensions of immunity (autoantibody specificity/effector quality vs. total immunoglobulin burden vs. systemic inflammation), their divergence does not exclude a biologically meaningful humoral surveillance phenotype. Nevertheless, our data were observational and do not establish causality; mechanistic interpretations should be regarded as hypothesis-generating. Prior reports—often cross-sectional or with ANA measured near or after cancer diagnosis—have described ANA positivity in association with higher HCC risk or more advanced disease in selected settings, underscoring that the meaning of ANA likely depends on timing, etiology, and immune context (13, 29). We are pursuing mechanistic work to determine whether and how ANA-linked humoral responses contribute to immune surveillance in cirrhosis; in that context, humoral recognition of altered self (e.g., ANA binding exposed nuclear antigens on dysregulated, hyper-replicating hepatocytes) could opsonize targets, activate complement, recruit Kupffer cell Fc/complement receptor phagocytosis, and engage NK-cell antibody-dependent cell-mediated cytotoxicity (ADCC), thereby preferentially clearing dysregulated clones while sparing quiescent hepatocytes via classic pathway complement and Fc/complement receptor clearance by Kupffer cells, and CD16-mediated NK-cell ADCC (30). We acknowledge that prior studies, largely cross-sectional or with ANA measured proximate to or after cancer diagnosis, have linked ANA positivity to higher HCC risk or more advanced disease, especially in HCV cohorts and historic series in which ANA titers rose as chronic liver disease progressed to HCC (29). These observations are important context for our findings and underscore the need for careful temporal anchoring of exposure and outcome. Our design differs in that ANA exposure was time-aligned to the risk window, outcomes were IRB-adjudicated at a high-volume center, and analyses leveraged a long calendar window (2005–2024), reducing reverse causation and chart-based misclassification while enhancing generalizability across changing etiologic patterns.

Importantly, heterogeneity in immune response and effector quality likely governs who mounts tumor-restraining autoantibodies. Factors such as immunogenetics, IgG subclass, and Fc glycosylation that alter complement activation and NK-cell engagement, degree and context of hepatocyte injury, NK-cell competence versus exhaustion within the cirrhotic microenvironment, etiology-specific antigens, and immunosuppression/exposures make uniform “protective ANA” biologically unlikely across all hosts (26). Our findings support a hypothesis-generating, quality-improvement framework in which ANA is treated as a time-stamped risk modifier, not a stand-alone decision rule. ANA positivity, even though associated with lower HCC risk in this cohort, should be interpreted as “lower risk but not exculpatory”: these patients should remain on guideline-concordant surveillance schedules, without lengthening intervals or downgrading imaging based on ANA alone. ANA-negative status is more ambiguous because it may reflect either truly quiescent biology or an immune-silent, higher-risk environment. For ANA-negative patients, we propose a conservative two-signal rule that combines ANA status with (i) background risk (fibrosis stage, adequacy of etiology control, metabolic/inflammatory activity, and degree of immunosuppression) and (ii) dynamic biomarker/imaging signals (AFP level or trajectory, liver enzymes, noninvasive fibrosis measures such as liver stiffness, and LI-RADS outcomes). When an ANA-negative patient also has high-risk features in either domain, intensified surveillance is reasonable (shorter recall intervals, proactive outreach to ensure adherence, and a low threshold for cross-sectional imaging). When both domains remain low risk over time, standard guideline surveillance is appropriate but should not be deintensified based on ANA negativity alone.

### Limitations

This study is observational, so residual and unmeasured confounding cannot be excluded despite prespecified adjustment for age, sex, liver disease severity (MELD and CTP), AFP, race/ethnicity, prednisone use, and etiology indicators. Residual confounding related to metabolic risk factors (e.g., body mass index/obesity, diabetes, and smoking) is possible, particularly within MASLD, because these variables were not collected as structured covariates in our dataset. Differences in health status, surveillance adherence, care access, or unrecorded immunomodulatory medications could influence both ANA measurement and downstream HCC detection. Although we anchored follow-up at the date of cirrhosis diagnosis and required at least 180 days of at-risk time to reduce reverse causation, subclinical cancers present near index or differential early surveillance could still bias associations. In addition, exploratory analyses restricted to MASLD cirrhosis and to the SMA-tested subset involved relatively few HCC events, limiting precision and precluding firm causal inferences.

ANA testing was clinically obtained rather than protocolized, raising the possibility of selection related to testing practices. For patients without ANA testing in the exposure window, our IRB-approved data plan limited detailed longitudinal abstraction to those meeting eligibility for the analytic cohort, and the DEEP6 prescreen export provided only limited demographics. As a result, we could not estimate follow-up duration or HCC incidence among patients without ANA testing, and residual selection bias cannot be fully excluded. However, baseline comparisons (Supplementary Table S1) and inverse-probability–weighted sensitivity analyses (Supplementary Table S2) demonstrated that the ANA–HCC association was directionally and quantitatively similar after reweighting for demographic differences in testing, supporting robustness to measured selection into ANA testing.

Exposure ascertainment relied on real-world ANA testing. Although we used a standard positivity threshold (≥1:40) and examined ordered titers, interassay variability and temporal changes in laboratory methods may have introduced nondifferential misclassification that would be expected to bias effects toward the null. ANA was treated as a baseline exposure; time-updated serologies, fluorescence patterns, and other autoantibody specificities were not available and therefore could not be evaluated. Similarly, surveillance intensity was not protocolized. Although we adjusted for severity proxies and modeled time to event, incomplete capture of imaging cadence may leave room for surveillance bias. Also, ANA pattern-specific associations with HCC could not be robustly evaluated due to the small number of events within less common pattern categories.

Data completeness for immunologic covariates remained a limitation. “NLR was more often missing than IgG,” so complete-case sensitivity models were used, which reduces sample size and may limit precision. Importantly, eligibility required a confirmatory imaging or histology assessment of HCC status occurring ≥6 months after index; patients without such follow-up were excluded. This design decreases outcome misclassification but introduces selection based on follow-up availability and contributed to the reduction from the DEEP 6 cohort builder screen to the enrolled analytic cohort; patients lost to follow-up or receiving care outside the system may therefore be underrepresented.

Finally, this is a single-center cohort, and external validity may be limited by local case mix, referral patterns, and laboratory practices. Replication in diverse health systems, with standardized serology, protocolized surveillance, and time-updated immune measures, will be essential to confirm generalizability and to test whether ANA-informed risk stratification improves clinical outcomes.

### Conclusion

Baseline ANA positivity in cirrhosis is independently associated with lower incident HCC risk after adjustment and internal validation, adding discrimination beyond routine clinical factors. As a low-cost, readily available test, ANA has practical potential to refine risk stratification for HCC surveillance. Prospective validation and mechanistic studies should confirm performance, explore titer/pattern granularity, and test whether ANA-informed, risk-adapted surveillance improves outcomes and efficiency. ANA could be integrated into risk-stratified HCC surveillance, while mechanistic studies probe how humoral recognition of altered self-constrains hepatocarcinogenesis.

## Supplementary Material

Table S1 Confirmed cirrhosis cohort - ANA tested vs not tested

Table S2 IPW sensitivity (weighted Cox): ANA positivity and incident HCC

Table S3 Autoantibodies and incident HCC in MASLD cirrhosis (exploratory)

Table S4 Sensitivity analysis – complete-case multivariable Cox model with IgG and NLR.

Figure S1 HCC-free survival in MASLD cirrhosis by ANA status

## Data Availability

Deidentified participant data underlying this article, the full statistical analysis code, the study protocol, and the variable dictionary will be made available by the corresponding author upon request to the editors and peer-reviewers and to the community at the time of publication, subject to Cedars-Sinai IRB requirements and an appropriate data-use agreement.
