# Supplementary material for: Natural Autoantibodies Negatively Correlate with Hepatocellular Carcinoma Incidence in Cirrhosis
Source: Cancer Res Commun. 2026 May 15;6(5):1136–45. doi: 10.1158/2767-9764.CRC-26-0007 (PMC13176760; doi:10.1158/2767-9764.CRC-26-0007)
Supplement: Table S1 — Confirmed cirrhosis cohort - ANA tested vs not tested [file crc-26-0007_table_s1_suppst1.docx]

**Table S1.** Confirmed cirrhosis cohort - ANA tested vs not tested

| **Characteristic** | **ANA tested (n=1023)** | **ANA not tested (n=806)** | **Total (n=1829)** | **Std diff** |
| --- | --- | --- | --- | --- |
| **Mean Age, years (SD)** | 53.7 (10.9) | 51.5 (13.1) | 52.7 (12.0) | 0.19 |
| **Sex: Male, n (%)** | 587 (57.4%) | 482 (59.8%) | 1069 (58.4%) | -0.05 |
| **Race/Ethnicity** |  |  |  |  |
| Hispanic-White, n (%) | 447 (43.7%) | 304 (37.7%) | 751 (41.1%) | 0.12 |
| Non-Hispanic White, n (%) | 378 (37.0%) | 289 (35.9%) | 667 (36.5%) | 0.02 |
| Non-Hispanic Asian, n (%) | 65 (6.4%) | 45 (5.6%) | 110 (6.0%) | 0.03 |
| Non-Hispanic Black, n (%) | 52 (5.1%) | 69 (8.6%) | 121 (6.6%) | -0.14 |
| Non-Hispanic Other, n (%) | 81 (7.9%) | 99 (12.3%) | 180 (9.8%) | -0.15 |

ANA-tested patients were slightly older and differed in recorded race/ethnicity distributions; other clinical covariates and outcomes were not available in the DEEP6 pre-screen export.
