# Supplementary material for: Natural Autoantibodies Negatively Correlate with Hepatocellular Carcinoma Incidence in Cirrhosis
Source: Cancer Res Commun. 2026 May 15;6(5):1136–45. doi: 10.1158/2767-9764.CRC-26-0007 (PMC13176760; doi:10.1158/2767-9764.CRC-26-0007)
Supplement: Table S2 — IPW sensitivity (weighted Cox): ANA positivity and incident HCC [file crc-26-0007_table_s2_suppst2.docx]

**Table S2.** IPW sensitivity (weighted Cox): ANA positivity and incident HCC

| **Model** | **N (events)** | **HR for ANA positivity (95% CI)** |
| --- | --- | --- |
| **Primary multivariable Cox (unweighted)** | 1016 (100) | 0.33 (0.20-0.53) |
| **IPW-weighted multivariable Cox (selection for ANA testing)** | 1016 (100) | 0.32 (0.20-0.52) |

Cox models were fit as complete-case for covariates (n=1,016; 100 HCC events)
