# Supplementary material for: Natural Autoantibodies Negatively Correlate with Hepatocellular Carcinoma Incidence in Cirrhosis
Source: Cancer Res Commun. 2026 May 15;6(5):1136–45. doi: 10.1158/2767-9764.CRC-26-0007 (PMC13176760; doi:10.1158/2767-9764.CRC-26-0007)
Supplement: Table S3 — Autoantibodies and incident HCC in MASLD cirrhosis (exploratory) [file crc-26-0007_table_s3_suppst3.docx]

**Table S3.** Autoantibodies and incident HCC in MASLD cirrhosis (exploratory)

| **Exposure**  **(MASLD only)** | **n** | **HCC events** | **Person-years** | **HCC rate /100 PY (95% CI)** | **Unadjusted HR (95% CI) vs ref** | **Adjusted HR* (95% CI) vs ref** |
| --- | --- | --- | --- | --- | --- | --- |
| **ANA− (<1:40)** | 128 | 21 | 514.5 | 4.1 (2.5–6.2) | 1.00 (reference) | 1.00 (reference) |
| **ANA+ (≥1:40)** | 116 | 5 | 454.3 | 1.1 (0.4–2.6) | 0.26 (0.10–0.69) | 0.28 (0.10–0.77) |
| **SMA− (<20 or “Negative”)** | 141 | 13 | 528.4 | 2.5 (1.3–4.2) | 1.00 (reference) | 1.00 (reference) |
| **SMA+ (≥20)**** | 65 | 8 | 278.5 | 2.9 (1.2–5.7) | 1.14 (0.47–2.76) | 1.19 (0.41–3.46) |

*Adjusted for age (per 10 years), sex, MELD ≥15, CTP class B/C, AFP ≥20 ng/mL, chronic prednisone exposure, and race/ethnicity (Hispanic-White, Non-Hispanic Asian, Non-Hispanic Black, Non-Hispanic Other vs Non-Hispanic White), SMA models additionally adjust for ANA status.

**SMA analysis restricted to MASLD patients with SMA measured; SMA− includes titers <20 and “Negative”, SMA+ includes titers ≥20.
