# Supplementary material for: Natural Autoantibodies Negatively Correlate with Hepatocellular Carcinoma Incidence in Cirrhosis
Source: Cancer Res Commun. 2026 May 15;6(5):1136–45. doi: 10.1158/2767-9764.CRC-26-0007 (PMC13176760; doi:10.1158/2767-9764.CRC-26-0007)
Supplement: Table S4 — Sensitivity analysis – complete-case multivariable Cox model with IgG and NLR. [file crc-26-0007_table_s4_suppst4.docx]

**Table S4.** Sensitivity analysis – complete-case multivariable Cox model with IgG and NLR.

| **Variable** | **Adjusted HR* (95% CI)** | **p value** |
| --- | --- | --- |
| **ANA positive (≥1:40) vs negative (<1:40)** | 0.45 (0.26–0.77) | 0.004 |
| **Age (per 10 years)** | 2.26 (1.66–3.07) | <0.001 |
| **Male sex** | 1.17 (0.70–1.95) | 0.555 |
| **MELD ≥15 (vs <15)** | 1.68 (0.95–2.98) | 0.073 |
| **CTP B/C (vs A)** | 0.89 (0.49–1.62) | 0.707 |
| **AFP ≥20 ng/mL (vs <20)** | 2.73 (1.38–5.38) | 0.004 |
| **Chronic prednisone use** | 0.59 (0.17–2.06) | 0.406 |
| **IgG (per 1 SD higher)** | 1.54 (1.20–1.98) | <0.001 |
| **Race/ethnicity** |  |  |
| Hispanic-White vs Non-Hispanic White | 0.87 (0.51–1.48) | 0.618 |
| Non-Hispanic Asian vs Non-Hispanic White | 0.75 (0.30–1.86) | 0.532 |
| Non-Hispanic Black vs Non-Hispanic White | 0.23 (0.03–1.78) | 0.158 |
| Non-Hispanic Other vs Non-Hispanic White | 0.47 (0.11–1.99) | 0.303 |
| **Etiology (vs all other etiologies)** |  |  |
| Autoimmune | 0.44 (0.10–1.90) | 0.269 |
| Multifactorial | 0.46 (0.14–1.54) | 0.207 |

Estimates are from a single multivariable cause-specific Cox model in the subset with complete data for IgG and differential counts used to compute NLR (n = 766; 75 HCC events), including all variables shown plus log-transformed NLR (per SD). NLR was not independently associated with HCC (HR 1.13, 95% CI 0.87–1.46; p=0.376) and is therefore not listed in the table. IgG and log (NLR) were standardized (z-scored) before modeling. Race/ethnicity reference is Non-Hispanic White. Multifactorial etiology denotes >1 documented contributing etiology (e.g., MASLD plus ALD and/or viral hepatitis).
