# Supplementary figures and images for: Natural Autoantibodies Negatively Correlate with Hepatocellular Carcinoma Incidence in Cirrhosis
Source: Cancer Res Commun. 2026 May 15;6(5):1136–45. doi: 10.1158/2767-9764.CRC-26-0007 (PMC13176760; doi:10.1158/2767-9764.CRC-26-0007)

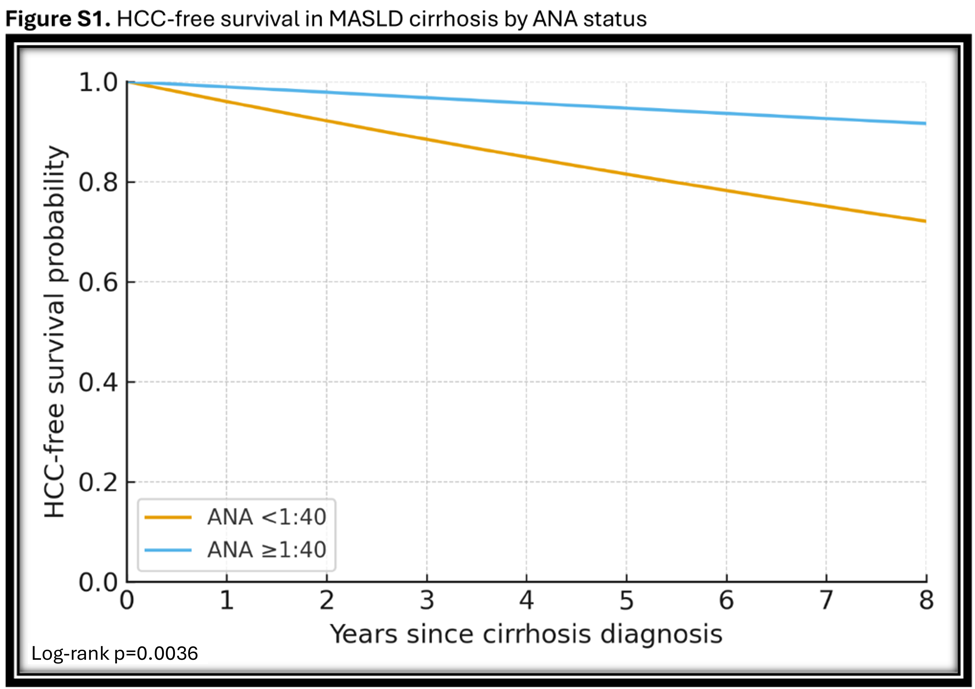

Supplement: Figure S1 — HCC-free survival in MASLD cirrhosis by ANA status [file crc-26-0007_figure_s1_suppsf1.png]
